# Effect of pH and Carbon Source on Phosphate Solubilization by Bacterial Strains in Pikovskaya Medium

**DOI:** 10.3390/microorganisms11010049

**Published:** 2022-12-23

**Authors:** Ma. Esther Sanchez-Gonzalez, Martha E. Mora-Herrera, Arnoldo Wong-Villarreal, Nadia De La Portilla-López, Laura Sanchez-Paz, Jorge Lugo, Rocio Vaca-Paulín, Pedro Del Aguila, Gustavo Yañez-Ocampo

**Affiliations:** 1 Laboratory of Edaphology and Environment, Faculty of Sciences, Autonomous University of the State of Mexico, Toluca 50000, Mexico; 2 Tenancingo Universitary Center, Autonomous University of the State of Mexico, Toluca 50000, Mexico; 3 Agrifood Division, Technological University of the Forest, Ocosingo 29950, Mexico

**Keywords:** *Bacillus pumilus*, biofertilizer, soluble phosphorus, Pikovskaya medium, sucrose

## Abstract

Phosphate-solubilizing bacteria (PSB) transform precipitated inorganic phosphorus into soluble orthophosphates. This study evaluated the efficiency of tricalcium and iron phosphate solubilization in Pikovskaya medium using five bacterial strains (A1, A2, A3, A5, and A6) cultured in acidic and alkaline pH levels. The bacterial strain that proved to be more efficient for P solubilization and was tolerant to pH variations was selected for assessing bacterial growth and P solubilization with glucose and sucrose in the culture medium. The bacterial strains were identified through 16S rRNA gene sequencing as *Pseudomonas libanensis* A1, *Pseudomonas libanensis* (A2), *Bacillus pumilus* (A3), *Pseudomonas libanensis* (A5), and *Bacillus siamensis* (A6). These five bacterial strains grew, tolerated pH changes, and solubilized inorganic phosphorus. The bacterial strain A3 solubilized FePO_4_ (4 mg L^−1^) and Ca_3_(PO_4_)_2_ (50 mg L^−1^). P solubilization was assayed with glucose and sucrose as carbon sources for A3 (*Bacillus pumilus* MN100586). After four culture days, Ca_3_(PO_4_)_2_ was solubilized, reaching 246 mg L^−1^ with sucrose in culture media. Using glucose as a carbon source, FePO_4_ was solubilized and reached 282 mg L^−1^ in six culture days. Our findings were: *Pseudomonas libanensis*, and *Bacillus siamensis,* as new bacteria, can be reported as P solubilizers with tolerance to acidic or alkaline pH levels. The bacterial strain *B. pumilus* grew using two sources of inorganic phosphorus and carbon, and it tolerated pH changes. For that reason, it is an ideal candidate for inorganic phosphorus solubilization and future production as a biofertilizer.

## 1. Introduction

Phosphorus (P) is a macronutrient that is present in all living things in its organic form as phytates, nucleic acids, and phospholipids. However, its bioavailability is very limited because in its inorganic form, and in an acidic media, it is found as iron or aluminum phosphate, and in an alkaline media, it is found as calcium phosphate [1,2]. 

In nature, P may be bioavailable through the action of phosphate-solubilizing bacteria (PSB) [3]. PSB have a key role in the P cycle as they make available precipitated P through the production of low-molecular-weight organic acids (i.e., malonic, gluconic, acetic, and lactic acid), which chelates metallic cations such as Ca^+2^, Fe^+3^, or Al^+3^, releasing orthophosphates as a result [4].

The bacterial solubilization of calcium phosphate is the most widely studied assay, and there are few studies exploring iron phosphate solubilization and even less at different pH ranges. Currently, the isolation of PSB is of major interest issue due to their potential use as biofertilizers, which could reduce the use of agrochemicals [5]. To consider a bacterial strain as biofertilizer for P solubilization, it is desirable for it to have good adaptation for growing in a wide range of pH values and the ability to solubilize several inorganic P sources [6].

PSB are heterotrophic microorganisms, and, therefore, to produce a bacterial biomass and enhance phosphate solubilization, there is a need to compare of the usage of different organic carbon sources in its culture media such that the bacterial strains show more growth [7]. The level of microbial activity for phosphate solubilization depends on the organic carbon degradation that sustains the PSB growth. This dependency on carbon degradation for PSB growth has not yet been studied nor reported widely. It is considered a key methodological step for PSB industrial production and application under field conditions to ensure their good performance [8]. 

The aims of this study were to evaluate five PSB strains through comparing their inorganic phosphorus solubilization under acidic and alkaline pH conditions, as well as to assess the effect of glucose and sucrose on their growth and P-solubilization.

## 2. Materials and Methods

### 2.1. Bacterial Strains Collection 

Five phosphate solubilizer bacterial strains (PSB) were isolated from soil surface samples (at first, 15 to 20 cm in depth) from agricultural soils where potatoes (*Solanum tuberosum*) are grown at Toluca’s Nevado in the Municipality of Zinacantepec, State of Mexico, Mexico (GPS: longitude (dec): - 99.805278, latitude (dec): 19.161389). The PSB screening and isolation were as reported by Yañez-Ocampo et al. [9].

A plate assay method was performed on Pikovskaya (PVK) agar [10] as follows: glucose, 10 g; (NH_4_)_2_SO_4_, 0.5 g; NaCl, 0.2 g; KCl, 0.2 g; MgSO_4_·7H_2_O, 0.1 g; MnSO_4_·7H_2_O, 0.5 g; FeSO_4_·7H_2_O, 0.5 g; yeast extract, 0.5 g; and 15 g of agar in 1 L of distilled water (pH 7.2); with the addition of 3.0 g of tricalcium phosphate as an insoluble P source. All reagents used were of analytical grade. The bacterial strains were selected by the formation of a clear halo around the bacterial colony after five incubation days at 28 °C in PVK agar. The selected isolates were kept using pure cultures and stored at 4 °C.

### 2.2. Phosphate Solubilization into Pikovskaya Liquid Medium

The liquid medium assays used 25 mL flasks with 10 mL of PVK medium and 3% (*v*/*v*) of the inoculum. Only the control flasks with sterile PVK liquid medium and without biomass were considered. The experiments were performed for 8 days at 28 °C and with 100 rpm agitation, after which measurements of the biomasses, orthophosphates, and pH levels were completed. The biomasses were measured spectrophotometrically by optical density at a wavelength of 600 nm. For determination of the orthophosphates, 5 mL aliquots of PVK medium were centrifuged to 1000× *g* for 25 min to obtain a biomass-free supernatant. The orthophosphates released in the PVK medium were determined using the molybdenum blue method [11,12], measuring the absorbance at a wavelength of 880 nm and converting this to concentration units using a standard curve. The pH levels of the PVK medium were recorded with a Multi 9620 IDS WTW^®^ model pH meter. All measurements were recorded from three replicates, respectively. 

### 2.3. Comparison of Phosphate Solubilization Efficiency by Bacterial Strains Cultured at Different pH Values 

All five PSB bacterial strains were cultured in PVK medium with different pH levels considering two pH ranges: an acidic range (5 and 5.5 pH) using FePO_4_ (100 mg L^−1^) as a non-soluble source of inorganic phosphorus and an alkaline range (7.5 and 8.0 pH) using Ca_3_(PO_4_)_2_ (100 mg L^−1^) as a non-soluble source of inorganic phosphorus. The pH levels were adjusted with 0.1 M HCl and 0.1 M NaOH, respectively. Each experimental treatment had six replicates. The experiments were performed using flasks with 10 mL of PVK broth inoculated with 300 µL of bacterial inoculum (the biomasses were adjusted to 0.2 absorbance units at a wavelength of 600 nm). The control flasks were not inoculated. All experimental treatments were incubated at 28 °C for 8 days. The biomass, orthophosphate, and pH quantification techniques used on the PVK liquid medium were the same as those mentioned in the above section.

### 2.4. Bacterial Strain Identification by 16S rRNA Gene Sequencing Using Bioinformatic Tools 

Five PSB bacterial strains were identified using a 16S rRNA gene-based molecular analysis method. The genomic DNA of each bacterial strain was extracted using a ZR Fungal/Bacterial DNA Kit TM. The 16S rRNA gene was amplified using the rD1 and fD1 oligonucleotides [13]. The amplification products were purified from electrophoresis gel using the GeneJET kit (Thermo Scientific, Waltham, MA, USA) and sequenced at the Institute of Biotechnology (IBT, Hong Kong) of the National University of Mexico (UNAM, Mexico City, Mexico). The obtained sequences were deposited in the GenBank of the National Center for Biotechnology Information (NCBI, Bethesda, MA, USA) with the following access numbers: (A1) ON679588, (A2) ON679589, (A3) MN100586, (A5) ON679590, and (A6) ON679591. The final sequences of the 16S ribosomal gene of each strain were compared with the 16S ribosomal genes deposited in the GenBank database using the BlastN bioinformatic tool. A matrix phylogenetic analysis was performed using the Kimura 2-parameter model with 1000 bootstrap using the Seaview 4.6.1 program [14]. 

### 2.5. Experimental Design to Assess the Bacterial Growth Optimization and Phosphate Solubilization by Comparing Two Carbon Sources

One bacterial strain was selected for its ability to tolerate both acidic and alkaline culture conditions. After the strain selection, the effect of two carbon sources (glucose and sucrose) in the culture medium was assessed with three factors: (1) using non-soluble phosphate sources (Ca₃(PO₄)_2_ and FePO₄) at initial concentrations of 1 g L^−1^; (2) using organic carbon sources (glucose and sucrose); and (3) days of incubation. The experiments featured factorial design. The treatments were: glucose plus tricalcium phosphate (GCa), sucrose plus tricalcium phosphate (SCa), glucose plus iron phosphate (GI), and sucrose plus iron phosphate (SI), and each one was performed in triplicate. A non-inoculated control was included in the experiments. Flasks with PVK liquid medium were inoculated with 300 µL of bacterial biomass (adjusted to 0.2 OD_600_) and incubated at 28 °C for 8 days. The pH variation, orthophosphates released, and biomass growth were quantified using the techniques mentioned in Section 2.2. All parameters were monitored at 0, 2, 4, 6, and 8 days.

### 2.6. Statistical Analysis

The phosphate solubilization results at both pH ranges (acidic and alkaline) for all five bacterial strains were analyzed using an ANOVA test and Tukey´s test. To optimize the bacterial growth and P-solubilization efficiency in the culture medium, two organic carbon sources and two non-soluble inorganic phosphorus sources were compared with a MANOVA test. Finally, the significant differences between treatments were observed using a Tukey´s test. A confidence level of 95% was applied in all experiments performed. Results were analyzed with the StatGraphics Centurion XV software.

## 3. Results and discussion 

### 3.1. Phosphate Solubilization by Bacterial Strains Cultured in PVK Liquid Medium at pH Levels of 5.0 and 5.5

During this work, five bacterial strains were capable of solubilizing both calcium and iron phosphates in alkaline and acidic pH environments, respectively. Table 1 shows the iron phosphate solubilization in an acidic pH range (5.0 and 5.5). At a pH of 5.0, the bacterial strain A1 showed significant differences in solubilization (4.420 mg L^−1^) compared to the other four bacterial strains. However, at a pH of 5.5, the bacterial strain A5 solubilized 4.722 mg L^−1^, and this result was statistically different from all the other bacterial strains tested. The solubilization of iron phosphates was reported by Collavino and Sansberro [15] where *Stenotrophomonas maltophilia* solubilized 12 mg L^−1^. Likewise, Hernández–Leal et al. [16], using edaphic bacteria, found an iron phosphate solubilization of 4.28 mg L^−1^. Chen et al. [17] reported that *Pantoea dispersa* solubilized iron phosphate at 41.42 mg L^−1^. Finally, Anzuay et al. [18] reported iron phosphate solubilization values of 14.5 and 37.4 mg L^−1^ using different strains of the *Serratia* genus. These results suggest that the low outcomes were due to the P source since iron phosphate has a solubility of 1.78 x 10^−9^ g L^−1^ and, therefore, bacterial activity could be limited.

A decrease in the pH of the culture medium was observed, which could be indirect evidence of the presence of organic acids. Initially, the pH of the culture medium was adjusted to 5.0 and 5.5, and after 8 days incubation time and bacterial growth, the pH was decreased to the range of 3.5 and 4.4, respectively. There have been some reports where the acidification of culture medium is due to organic acids being produced by the PSB [19,20], as this is the main microbial mechanism for the solubilization of inorganic P [21]. Anzuay et al. [18] reported that culture medium acidification could have negative effects on P solubilization, suggesting that when there are pH decreases of 4.5 and 3.5, FePO_4_ solubility also decreases. 

### 3.2. Phosphate Solubilization by Bacterial Strains Cultured in PVK Liquid Medium at pH levels of 7.5 and 8.0

After assessing P solubilization in an acidic pH range, we performed assays in an alkaline pH range (7.5 and 8.0) using tricalcium phosphate as a non-bioavailable P source. The highest phosphate solubilization value was 27.406 mg L^−1^, which was achieved at a pH of 7.5 and obtained by the bacterial strain A3 (Table 2). However, the bacterial strain A2 had significantly higher results, solubilizing 52.319 mg L^−1^ at a pH of 8.0. All bacterial strains showed better phosphate solubilization values at a pH of 8.0 than at a pH of 7.5. It is very remarkable that the concentration of solubilized P nearly doubled. All bacterial strains assayed showed better phosphate solubilization performances in the alkaline pH range than in the acidic pH range; therefore, tolerance was observed in their biological activities for both pH ranges. The solubilization of Ca_3_(PO_4_)_2_ has been reported in multiples studies. Ogut et al. [22] reported that *Acinetobacter* sp. WR326 solubilized 888 mg L^−1^ of Ca_3_(PO_4_)_2_. Another study by Prieto-Correal et al. [23] showed that *Streptomyces* T3A solubilized 600 mg L^−1^ of Ca_3_(PO_4_)_2._

Pande et al. [24] found that *Burkholderia cepacia* was able to solubilize 305 mg L^−1^ Ca_3_(PO_4_)_2_. Sowmya et al. [25] reported that Ca_3_(PO_4_)_2_ solubilization for the *Pantoea* and *Enterobacter* genus was 500 mg L^−1^ and 1200 mg L^−1^, respectively. The variations found in tricalcium phosphate solubilization rates could be associated with the metabolic characteristics of the bacteria analyzed. Prieto-Correal et al. [23], as a possible explanation for the low values of soluble phosphorus, posited that the phosphorus might be metabolized and immobilized as part of the microbial biomass. This may also explain the results of our research.

### 3.3. Bacterial Growth in PVK Liquid Medium in Both Acidic and Alkaline pH Ranges

**Figure 1:**
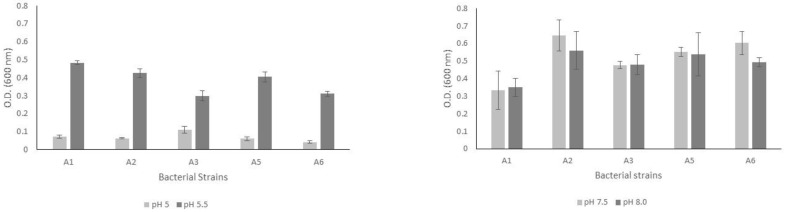
Results of bacterial growth measured by optical density for the five bacterial strains cultured in PVK medium in both acidic (**left** graph) and alkaline (**right** graph) pH ranges.

After eight culture days, the bacterial strains grew in both acidic and alkaline pH ranges (Figure 1). The bacterial biomasses reached 0.1 absorbance units when cultured at a pH of 5.0. In contrast, we observed higher growth rates of the biomasses when the bacterial strains were cultured at a pH of 5.5. Moreover, the five bacterial strains analyzed showed better adaptability for growing in an alkaline environment since the biomasses’ values were between 0.3 and 0.5 absorbance units. 

### 3.4. Bacterial Strains Identification and Properties Reported

Table 3 shows the molecular identification results of the 16S ribosomal gene analysis, which were obtained using the sequences of the five bacterial strains. After BLAST analysis, it was observed that strains A1, A2, and A5 had more similarities with species of the *Pseudomonas* genus, while the bacterial strains A3 and A6 showed a relationship to the *Bacillus* genus. 

**Figure 2:**
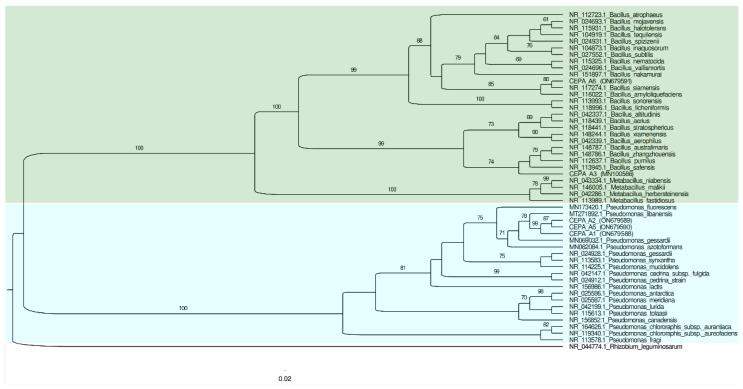
Phylogenetic tree of the 16S rRNA gene sequencing for all five bacterial isolates with the ability to solubilize phosphate. The reference sequence accession numbers are shown after the species name.

The phylogenetic tree was built using the 16S ribosomal gene sequences fragments to confirm the identities of the isolates at the genus level (Figure 2). The cladogram showed that the A1, A2, and A5 strains were related to *Pseudomonas libanensis*. These bacteria have been recently identified and reported as drought stress alleviators in wheat cultures grown under greenhouse conditions [26].

Meanwhile, bacterial strain A3 was 96.03% related to *Bacillus pumilus,* and bacterial strain A6 was 99.07% related to *Bacillus siamensis*. Recently, *Bacillus pumilus* was recognized as a plant growth promoter rhizobacteria capable of synthesizing phytohormone precursors while also having an antagonistic activity against nematodes [27], in addition to chitinase activity in association with rice crop roots, enhancing carbohydrate metabolism and phenylpropanoid biosynthesis [28] and being a drought stress alleviator [29]. The bacterial species *B. siamensis* has been reported as the endophyte bacterium of *Cicer arietinum* L., showing antifungal activities [30]. 

The primary reports related to phosphate solubilizing with bacteria are those from the *Bacillus* genus and, more frequently, *Bacillus subtillis*. In the study by Hanif et al. [31], *B. subtillis* solubilized phosphate and increased the length and weight of both the roots and shoots in potatoes grown in soils with low phosphorus content. Similar results were reported by Ahmad et al. [32], where several phosphate and zinc solubilizer bacterial strains were identified through 16S rRNA sequencing, including *Bacillus subtilis* IA6 (accession # MN005922), *Bacillus* sp. IA16 (accession # MN005924), and *Bacillus aryabhattai* IA20 (accession # MN005925). They concluded that these bacterial isolates have promising uses as inoculants for improving cotton growth under semi-arid conditions. 

Zaheer et al. [33] reported phosphorus solubilization by two bacterial isolates identified as *Pseudomonas* sp. strain AZ5 and *Bacillus* sp. strain AZ17. They also noted that both genera had positive effects on plants because of their multifunctional to P and Zn solubilization and production of IAA, phytase, organic acids, HCN, N-Acyl homoserine lactones, and siderophores, all of which are useful traits for plant growth. According to our results, *Pseudomonas libanensis*, *P. gessardii, Bacillus pumilus,* and *B. siamensis* are new contributions to the class of phosphate solubilizing bacteria (PSB).

### 3.5. Bacterial Growth and Phosphate Solubilization Comparison through the use of Glucose and Sucrose in PVK Culture Medium

**Figure 3:**
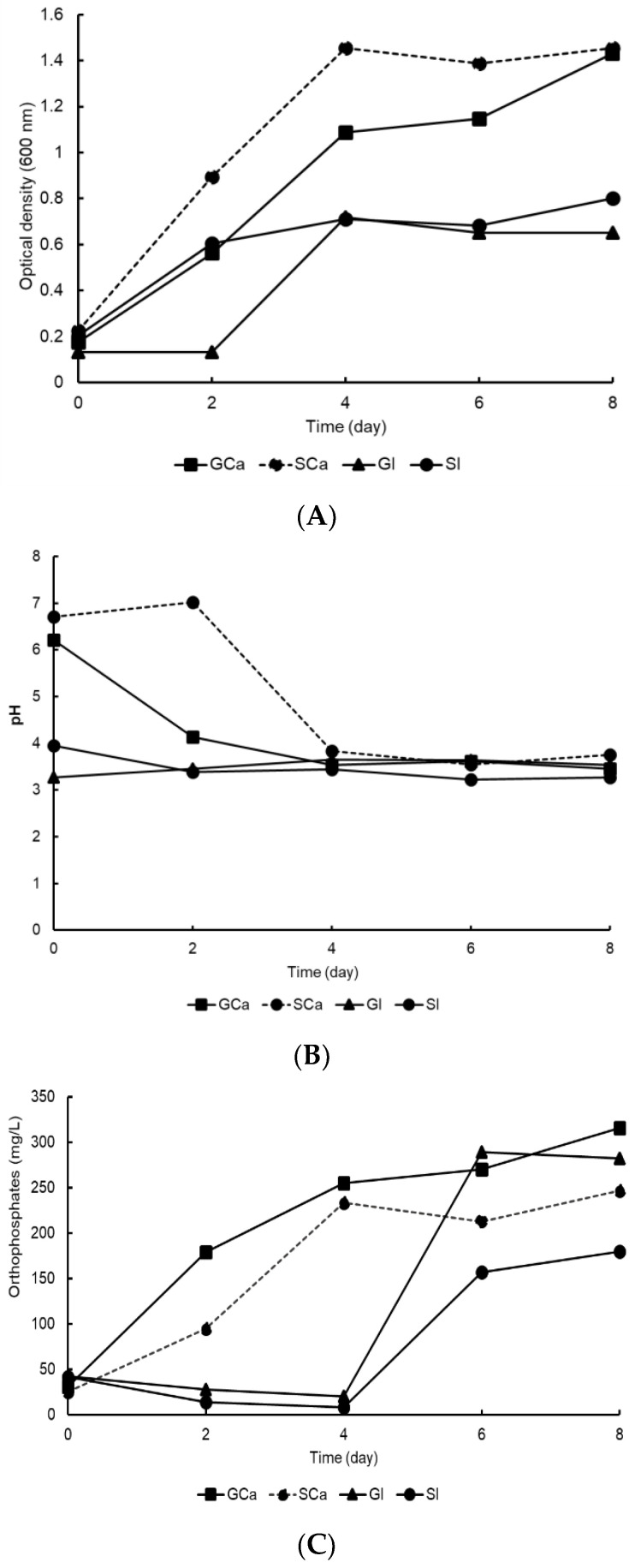
Growth, pH, and P solubilization by the A3 bacterial strain, with glucose or sucrose in PVK medium. (**A**) Bacterial growth measured by optical density. (**B**) Dynamics of pH values. (**C**) P solubilization.

The results obtained in this research showed that bacterial strain A3 was capable of growing and solubilizing tricalcium phosphate and iron phosphate as it could tolerate both acidic and alkaline pH levels. Therefore, it was selected to compare its growth and P solubilization using glucose or sucrose in PVK medium as the main carbon source. Figure 3A shows that bacterial strain A3 grew in a PVK culture medium with both sources of carbon and phosphorus. In the PVK medium with added sucrose and tricalcium phosphate, the A3 biomass increased from 0.2 to 1.4 absorbance units. In contrast, the A3 cultured with iron phosphate and sucrose or glucose grew to only half of that treated with tricalcium phosphate. 

The behavior of pH throughout the experiment is reported in Figure 3B. The pH values at the beginning of the experiment in the treatments with tricalcium phosphate (GCa and SCa) tended towards neutrality. In the iron phosphate treatments (GI and SI), the initial pH values were acidic, and this remained constant throughout the experiment. After two culture days, the acidification process had initiated only in the treatments with tricalcium phosphate, indirectly indicating the production of organic acids by the A3 bacterial strain.

The solubilization of tricalcium phosphate began after two days of incubation, which was the same timeframe as the acidification process. The solubilization of iron phosphate did not begin until the fourth day of the experiment (Figure 3C). The highest soluble phosphorus values were recorded in the glucose treatments (GCa (315 mg L^−1^) and GI (289 mg L^−1^)). In the sucrose treatments with calcium (SCa) and iron (SI) phosphate, the soluble phosphorus values were 246 mg L^−1^ and 179 mg L^−1^, respectively. 

Acidification is a key step towards Ca_3_(PO_4_)_2_ solubilization. Bacteria dissolve calcium phosphates through the production of gluconic and 2-ketogluconic acids through enzymes located in the periplasmic space [17]. Sowmya et al. [25] reported that there was a direct relationship between P solubilization and medium acidification. It has been reported that acidification modifies the precipitation/dissolution equilibrium of P and calcium sequestration by organic acids, and this results in P solubilizing and bioavailability [5,7]. Aliyat et al. [34] recently showed the decreasing pH of culture medium through organic acids production (formic, propionic, isobutyric, butyric, isovaleric, caproic, and heptanoic acids) by PSB strains. Benbrik et al. [19] pointed out that in a liquid culture medium, microbial biomass respiration generates CO_2,_-producing carbonic acid, creating acidic conditions and, consequently, bacterial growth inhibition, as well solubilization activity. 

Mukhtar et al. [35] and McRae and Monreal [36] noted that PSBs use glucose and sucrose as carbon sources because these are the predominant sugars in rhizosphere exudates. Stefaoni et al. [37] noted that glucose increases solubilizing activity by serving as a substrate for the synthesis of gluconic acid. This acidifies the medium, allowing the phosphate solubilization. Glucose also favors the synthesis of oxalic acid, a key element for solubilizing Ca_3_(PO_4_)_2_, by acting as a calcium chelator.

Lim et al. [38] and Shen et al. [39] reported that the solubilization of phosphates is related to the physiology, metabolism, and genetic constitution of each bacterial species, and depending on the time required for solubilizing inorganic P, PSBs can be classified into early and late solubilizers, with early solubilizers being those having a maximum P-solubilization value at 72 incubation hours and late solubilizers being those that solubilize P after four or five incubation days. According to this, the A3 bacterial strain could be considered a late solubilizer since it solubilized tricalcium and iron phosphate on the eighth and sixth incubation days, respectively.

## 4. Conclusions

The main findings of this research were that the bacterial strains here studied were tolerant to alkaline or acidic conditions and retained their phosphate solubilization activity. This is an important outcome because it demonstrates that these strains can be applied as biofertilizers in soils with similar pH levels. The bacteria *Pseudomonas libanensis,* and *Bacillus siamensis* were novel findings for their phosphate solubilization activities. Finally, *Bacillus pumilus* became an ideal candidate for promoting sustainable agriculture through its application as biofertilizer due to its proven ability to grow in both pH ranges, its metabolic versatility in using different carbon sources, and its efficiency in solubilizing iron phosphate and tricalcium phosphate.

## Figures and Tables

**Table 1:** Phosphate solubilization by PSB in acidic pH regions after 8 days of incubation.

	pH 5		pH 5.5	
Bacterial Strains	Orthophosphates (mg L^−1^)	pH	Orthophosphates (mg L^−1^)	pH
**A1**	4.420 ± 1.802 ^a^	3.50 ± 0.13 ^a^	3.370 ± 0.432 ^a^	4.13 ± 0.04 ^b^
**A2**	2.826 ± 1.035 ^ab^	3.50 ± 0.03 ^a^	3.792 ± 1.079 ^a^	4.12 ± 0.03 ^b^
**A3**	2.585 ± 0.952 ^ab^	3.45 ± 0.04 ^a^	3.297± 0.503^a^	4.06 ± 0.04 ^b^
**A5**	2.560 ± 0.830 ^ab^	3.51 ± 0.10 ^a^	4.722 ± 1.692 ^a^	4.15 ± 0.11 ^b^
**A6**	2.488 ± 0.747 ^b^	3.61 ± 0.20 ^a^	1.087 ± 0.392 ^b^	4.43 ± 0.03 ^a^

These data represent averages ± means and standard deviations. The superscript letters denote significant differences between the bacterial strains determined by Tukey´s tests (*p* < 0.05).

**Table 2:** Phosphate solubilization by PSB in alkaline pH regions after 8 days of incubation.

	pH 7.5		pH 8.0	
Bacterial Strains	Orthophosphates (mg L^−1^)	pH	Orthophosphates (mg L^−1^)	pH
**A1**	22.561± 7.211 ^a^	3.22 ± 0.41 ^c^	38.200 ± 7.739 ^ab^	3.38 ± 0.09 ^a^
**A2**	23.971 ± 3.597 ^a^	4.26 ± 0.10 ^ab^	52.319 ± 10.772 ^a^	3.17 ± 0.18 ^b^
**A3**	27.406 ± 6.094 ^a^	4.31 ± 0.26 ^a^	50.435 ± 7.345 ^a^	3.08 ± 0.05 ^b^
**A5**	12.765 ± 5.314 ^b^	3.89 ± 0.03 ^b^	27.005 ± 10.792 ^b^	3.44 ± 0.12 ^a^
**A6**	7.947 ± 4.232 ^b^	3.96 ± 0.05 ^ab^	50.870 ± 12.831 ^a^	3.06 ± 0.10 ^b^

These data represent averages ± means and standard deviations. The superscript letters denote significant differences between the bacterial strains determined by Tukey´s tests (*p* < 0.05).

**Table 3:** Bacterial strains identified through 16S rRNA gene analysis.

Bacterial Strain	Possible Genus	Related Strains	Query Cover (%)	Identity (%)
A1	*Pseudomonas* sp.	*Pseudomonas libanensis*	100	98.58
A2	*Pseudomonas* sp.	*Pseudomonas libanensis*	100	95.36
A5	*Pseudomonas* sp.	*Pseudomonas libanensis*	99	98.48
A3	*Bacillus* sp.	*Bacillus pumilus*	98	96.03
A6	*Bacillus* sp.	*Bacillus siamensis*	100	99.07

## Data Availability

Not applicable.
